# Multitype Quantum Well Semiconductor Membrane External-Cavity Surface-Emitting Lasers for Widely Tunable Continuous Wave Operation

**DOI:** 10.1021/acsphotonics.3c01801

**Published:** 2024-08-25

**Authors:** Patrik Rajala, Philipp Tatar-Mathes, Hoy-My Phung, Jesse Koskinen, Sanna Ranta, Mircea Guina, Hermann Kahle

**Affiliations:** † Optoelectronics Research Centre (ORC), Physics Unit/Photonics, Faculty of Engineering and Natural Science, Tampere University, Korkeakoulunkatu 3, 33720 Tampere, Finland; ‡ Robert Bosch GmbH, Robert-Bosch-Campus 1, 71272 Renningen, Germany; § Department of Physics & Astronomy, The University of New Mexico, 210 Yale Boulevard NE, Albuquerque, New Mexico 87106 ,United States

**Keywords:** semiconductor laser, VECSEL, MECSEL, wavelength tuning, broadband gain, optical pumping, wide bandwidth, constant power

## Abstract

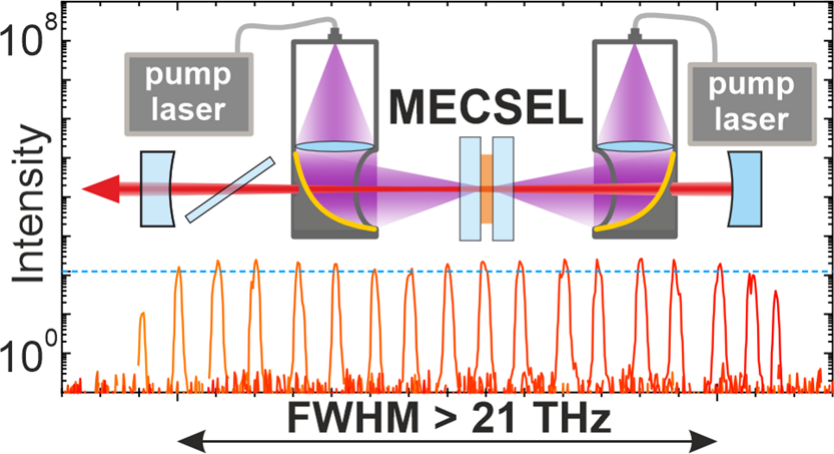

Membrane external-cavity surface-emitting lasers (MECSELs) represent a cutting-edge approach in pushing the performance boundaries of vertically emitting semiconductor lasers. The fundamental concept of employing an extremely thin gain membrane, spanning from hundreds of nanometers to a few micrometers in thickness and sandwiched between transparent heat spreaders, introduces novel opportunities through uniform double-sided optical pumping and enhanced heat dissipation from the active region. Additionally, these advantages of MECSELs facilitate more intricate band gap engineering possibilities for the active region by integrating multiple types of quantum wells (QWs) into a single laser gain structure. This work introduces a novel design strategy for laser gain structures incorporating various QW types. The objective is to achieve broad-spectrum gain with relatively high-power operation and potentially a flat spectral tuning range. Our design focuses on ensuring sufficient gain across a wide wavelength span, achieving uniform pump absorption, and limiting carrier mobility between different quantum well types during laser operation. We demonstrate a full-width half-maximum (FWHM) tuning range exceeding 70 nm (equivalent to more than 21.7 THz) with over 125 mW of output power across this entire tuning range at room temperature.

## Introduction

During the last decades, many application fields ranging from atomic and molecular physics^1^ to spectroscopy^2,3^ and from broadband sensors to optical telecommunication systems^4,5^ have shown an increasing demand for widely tunable lasers.^6^ Due to their compactness, low costs, and relatively high efficiencies, semiconductor lasers are in general favored over other laser systems. When it comes to highly tunable lasers, titanium-sapphire lasers (Ti:sapph.) are the gold standard. Their ability to produce a flat emission power spectrum on wavelengths ranging roughly from 650 to 1100 nm is superior to any semiconductor laser.^7,8^ However, many application fields mentioned above do not require this whole tuning range or the power level that Ti:sapph. provides. At the same time, they would benefit strongly from the unique properties of semiconductor lasers, particularly the compactness, ease of manufacturing, and lower costs, if these could be achieved while maintaining a relatively flat spectral tuning range. Membrane external-cavity surface-emitting lasers (MECSELs)^9,10^ are able to answer this need by enabling the use of advanced gain structures with multitype quantum well (QW) designs, as will be demonstrated in this paper.

**Figure 1:**
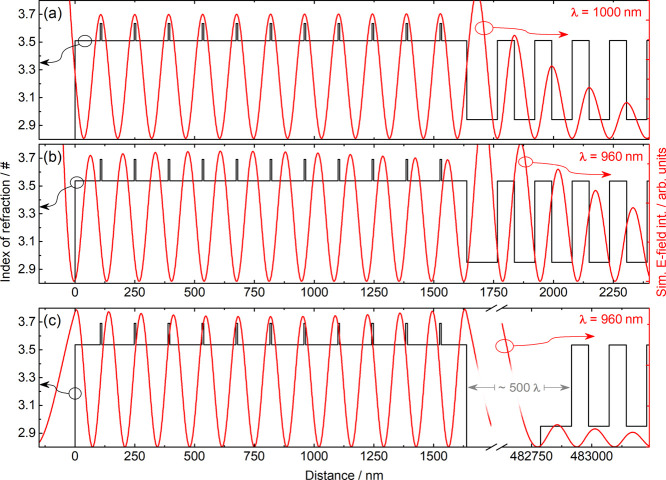
(a) Simplified exemplary VECSEL gain chip for operation at a 1 μm wavelength with simulated *E*-field intensity. The antinodes of the standing wave of the optical field match the QWs and resonant operation is present. (b) Exact same structure with simulated *E*-field intensity at 960 nm. The standing wave of the optical field no longer overlaps with the QWs, and the laser emission would stop as no field enhancement could be created. (c) Same active region but now in a MECSEL configuration with the external cavity mirror about 500 λ away from the active region membrane. The laser “finds” the best possible overlap between the standing wave of the optical field and the quantum wells, even though the match is no longer perfect.

Leinonen et al.^11,12^ have demonstrated a vertical external-cavity surface-emitting laser (VECSEL) capable of operating on two different wavelengths at the same time owing to the use of two different kinds of QWs in a single laser gain structure, which proves that the operation of a surface-emitting laser employing a relatively complex QW gain structure is certainly feasible. Wider tunability and operation on two different wavelengths with the aid of utilizing nonlinear optical processes, namely, second-harmonic generation (SHG) and sum-frequency generation (SFG), have been demonstrated more recently as well.^13^ Other strategies to achieve wide tuning ranges with VECSELs have included a multi-chip cavity configuration,^14^ the use of an inserted blade,^15^ a two-mode resonant microcavity,^16^ careful gain element reflectance engineering, and optimization of the laser gain element^17,18^ as well as the use of quantum dots (QDs)^19^ providing a broad gain bandwidth. However, the VECSEL technology used in all of these previous demonstrations imposes certain limits on continuous tuning throughout the wavelength range enabled by the QWs or QDs. The main limitation arises from the monolithically integrated distributed Bragg reflector (DBR), which locks the phase of the standing wave of the optical field in a VECSEL’s gain region during laser operation, as can be seen in Figure 1. Figure 1a demonstrates how the standing wave of the optical field in a VECSEL on the designed operation wavelength, which in our example is 1 μm, overlaps well with the QWs to create field enhancement. Figure 1b in turn demonstrates how changing the operation wavelength away from the design wavelength (demonstrated in Figure 1a) shifts the overlap between the standing wave of the optical field and the gain providing QWs completely out of phase because the DBR locks the phase of the optical field at its interface. In MECSELs, this limitation shown in Figure 1b is removed,^20^ as both mirrors are external and the phase of the optical field on the edges of the laser membrane is not predetermined, but rather depends on the current state of the external mirrors and the entire cavity. This gives the laser the ability to “find” the best possible overlap between the standing wave of the optical field and the gain providing QWs in any situation and provides enough gain for laser operation even on a wavelength that is far from the designed operation wavelength, as is illustrated in Figure 1c.

## Design and Structure Simulation

While the addition of more than one type of QWs to a MECSEL gain structure does not significantly complicate the epitaxial growth or the processing of the MECSEL, there are a few important additional steps in the design phase that need to be taken into account. The first demonstration of a multitype QW surface-emitting laser by Leinonen et al.^11^ had two leading design principles to realize a dual-wavelength VECSEL: (1) the use of electron blocking layers (EBLs) between different kinds of QWs to prevent the excited electrons and holes from only diffusing toward the structure’s lowest energy states, which would be the QWs of the nominally longer emission wavelength, and (2) making sure that during the shorter emission wavelength operation of the laser, the antinodes of the standing wave of the optical field overlap with the QWs emitting on that wavelength, while at the same time the nodes of the optical field overlap with the longer emission wavelength QWs. During the longer wavelength operation, the overlaps take place vice versa. When we now consider continuous broadband tuning operation instead of dual-wavelength operation, design point (1) is still important to take into account, as otherwise, the charge carriers will strongly diffuse toward the longer wavelength QWs’ energy states in all situations. This would significantly reduce the tuning range available, which was effectively demonstrated by Leinonen et al.^11^ with their photoluminescence (PL) measurement results. Point (2), however, is a bit more complicated for the cases of continuous tuning and using the MECSEL technology instead of VECSELs. On the shorter wavelength end, it is clearly beneficial that the shorter wavelength QWs match the antinodes of the standing wave of the optical field, while its nodes should match the QWs of the longer wavelength to limit the reabsorption of photons emitted from the QWs of the shorter wavelength during operation. However, the other way around, the reabsorption does not play a significant role, as the QWs of the shorter wavelength due to their higher band gap cannot reabsorb the photons emitted by the longer wavelength QWs. The absence of a phase-locking DBR allows the laser to choose the suitable phase on whatever wavelength it is currently being operated on, as depicted in Figure 1 (see also Tatar-Mathes et al.^21^). Thus, when operating the MECSEL on the longer wavelength end, it is enough to just ensure a good matching between the standing wave optical field’s antinodes and the longer wavelength QWs. This allows for an extra degree of freedom in the design process of a continuously tunable MECSEL.

In addition to the mentioned design points, significant aspects in the design process of the continuously tunable MECSEL are (1) the choice of QW materials, (2) the amount of QWs both per group and in total, and (3) the distribution of pump power inside the structure during operation. To choose the correct QW materials, the tunability of a single type of QW must be considered. A wide, full tuning range for VECSELs utilizing only a single type of QW in the wavelength range around 1 μm has been published, e.g., by Borgentun et al.^17,18^ with 32 and 43 nm, respectively, and by Broda et al.^16^ with 70 nm (without temperature tuning). The latest record values for MECSELs within the 1.0 to 1.2 μm range have been published by Priante et al.^22^ and Yang et al.^9,20^ In addition to the actual tuning range received from a single QW type, we must note from the mentioned results that the tuning range is not symmetrically distributed around the center emission wavelength, but it instead follows the typical inverted parabolic behavior, which, should the cavity mirrors possess a flat reflection characteristic, will reassemble the spectral shape of the gain above laser threshold. Thus, when choosing the QW materials for a multitype QW gain structure, a compromise between maximum tuning range and constant emission power everywhere within must be made. Based on previous experiments and the data mentioned, we chose a nominal emission wavelength separation of 60 nm between the two QW types in order to receive a relatively constant emission power distribution throughout the whole tuning range. In the 1 μm wavelength range that was chosen for demonstration purposes for this paper, adequate nominal wavelengths were then 950 and 1010 nm. 7 nm thick InGaAs QWs with In fractions of 13.0 and 20.5%, respectively, were used to achieve those nominal emission wavelengths. In addition, 10 nm thick GaAs barriers, GaAs spacers of varying thicknesses, 25 nm thick GaAsP strain compensation layers, 30 nm thick AlAs EBLs (transparent to both the pump and emission wavelengths), and 30 nm thick lattice-matched GaInP window layers were employed in the structure. The number of QWs per group that can be used is almost entirely dictated by the amount of local strain that the structure can withstand, and thus an amount of two QWs per QW group was chosen in our design.

The pump power absorption profile inside the structure during operation was taken into account when designing the total amount of QWs and their distribution. The shorter emission wavelength QWs (950 nm) have 33% less quantum confinement for holes and 37% less for electrons compared to the 1010 nm QWs. Thus, the 950 nm QWs provide less small-signal gain. This difference had to be taken into account in order to promote a more homogeneous output power distribution over the whole length of the tuning range, which was done by utilizing more shorter-wavelength QWs in comparison to longer-wavelength QWs in the final MECSEL structure. For the exact relations, however, the total thickness of the structure must be determined. Based on the simulations by Phung et al.,^23^ we derived that ∼2 μm is a suitable target for the total thickness of the structure, as we could then still optically pump all of the QWs sufficiently from one side of the MECSEL chip. We call this pumping regime single-side pumping (SSP). The order of the QW groups inside the structure was otherwise designed to take advantage of the unique possibility of MECSELs, double-sided pumping (DSP), but to be able to compare the changes between the different pumping regimes, the total thickness was chosen so that the structure can be operated in the SSP regime as well. Thus, the final structure thickness became 2.041 μm, which, after taking into account how thick the required strain compensation, barrier, charge carrier blocking, and spacer layers must be, allowed us to use 11 QW groups of two QWs per group. To accommodate the ∼35% difference in quantum confinement between the two types of QWs as closely as possible, we decided to distribute these 11 groups as 7 shorter wavelength QW groups and 4 longer wavelength QW groups.

When the amounts and specific materials of the layers as well as the total thickness of the structure were decided, the only thing left to do was to place all of them inside the structure accordingly. To make sure that both QW types receive a relatively even amount of pump power to be absorbed during DSP, the QW distribution was designed to be symmetric with respect to the middle point of the structure. To match the antinodes and nodes adequately during the different operation wavelengths, as described earlier in this section, the placements and thicknesses of the spacer layers were fine-tuned in the very last phase of the design process. This resulted in the final structure being not precisely symmetric but rather nearly so. The fine-tuning of the thicknesses of the spacer layers was done with the aid of a simulation of the standing wave of the optical field inside the structure during operation on both the shorter target wavelength of 950 nm and the longer target wavelength of 1010 nm. The final thicknesses of the spacer layers were determined by finding the best compromise between the three goals already mentioned at the beginning of this chapter: simultaneously matching each target wavelength’s optical field’s antinodes with their corresponding QWs, while also matching the nodes of the optical field on the longer emitting QWs when operating on the shorter target wavelength of 950 nm.

**Figure 2:**
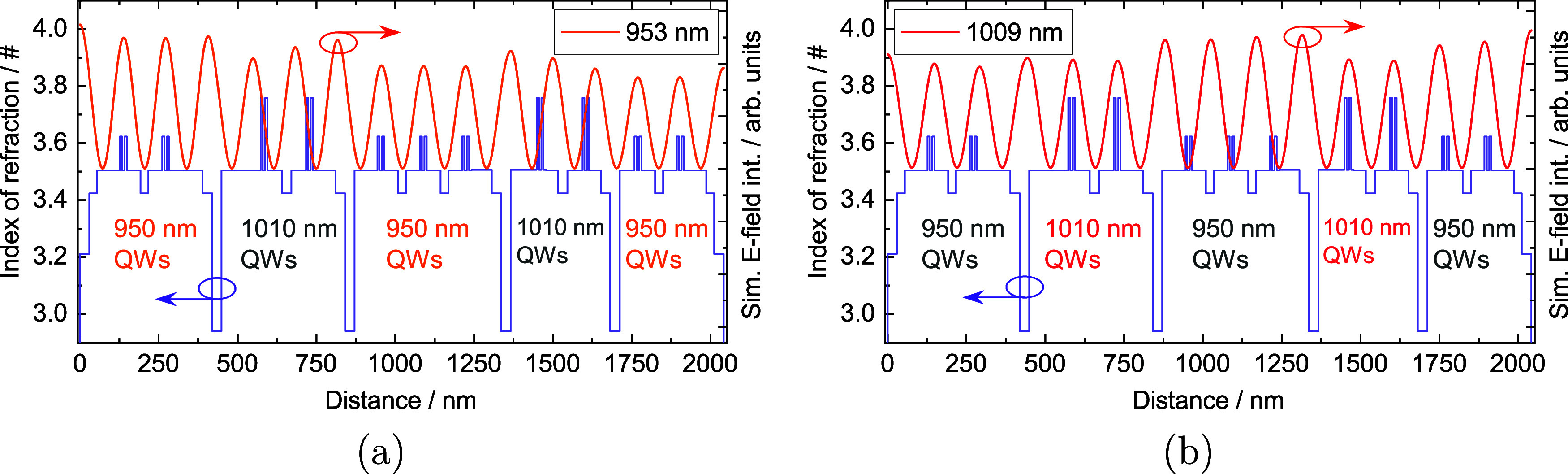
Gain membrane’s layer structure is shown. (a) Refractive index (violet solid line) and the simulated standing wave of the optical field intensity (solid orange line) of the short wavelength (nominally 953 nm) resonance are plotted over the thickness of the gain membrane. (b) Refractive index (violet solid line) and the simulated standing wave of the optical field intensity (solid red line) of the long wavelength (nominally 1009 nm) resonance are plotted over the thickness of the gain membrane.

The final structure design combining all of the design points mentioned in this section is shown in Figure 2. Figure 2a shows the simulated standing wave of the optical field intensity inside the structure during operation on the shorter wavelength of 953 nm, while Figure 2a shows the same simulation on the longer wavelength of 1009 nm. The optical field simulations were made utilizing the transfer matrix method and assuming a lasing temperature of 350 K, but for the active design structure process, we used the SimuLase software.

Figure 2a,b also shows the placements of the GaAsP strain compensation layers and the 11 QW groups that are divided into five sections by the EBLs mentioned at the beginning of this chapter. This kind of order and distribution of QW groups was chosen as a balance between (1) hitting the total structure thickness target of ∼2 μm, (2) keeping the structure simple enough to have the mentioned antinode and node matchings on different wavelengths, while (3) still having a similar amount of total pump light reaching each of the QW group types in both DSP and SSP regimes. Based on the Phung et al.^23^ simulations, we estimated that 33% of the pump light will be absorbed in the longer wavelength QW group sections and 62% in the shorter wavelength QW group sections in the structure depicted in Figure 2a,b. When taking into account the four to seven relation in the number of QW groups, both QW types receive a relatively even amount of pump light per QW in this design. While all of the critical design targets were reached with the balancing acts, they led to a situation where three antinodes (seen at distances ∼425, 850, and 1350 nm in Figure 2a,b) do not overlap with any of the QW pairs on either of the target wavelengths. Ultimately, this limitation arises from the need to accommodate the space for the EBLs.

The actual lasing tests were done by mounting the sample to the lasing setup (shown in Figure 4) in the orientation depicted in Figure 2. Since the barriers around the QWs are slightly thicker on the left side of the structure, we expected the performance to be slightly better when pumping the MECSEL from the right side during SSP measurements. The thicker barriers compensate for the smaller pump power density reaching the left side of the MECSEL when pumping from the right, which, in turn, leads to a more homogeneous pumping condition for all QWs, ultimately leading to a better performance. This is further discussed in the characterization results in the section “Broadband Wavelength Tuning”.

### Photoluminescence Characterization

**Figure 3:**
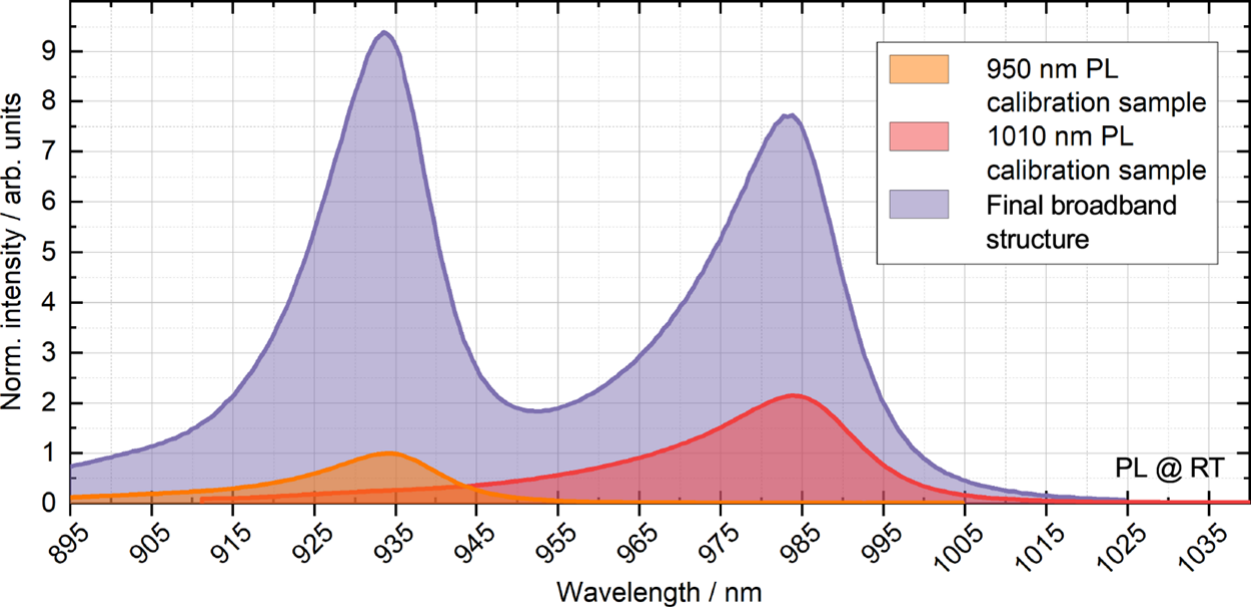
PL signal of the final broadband laser structure (violet) at room temperature (RT) compared to the PL of the 950 nm QW calibration sample (orange) and the 1010 nm QW calibration sample (red). Measurement was done with an RPM 2000 system using a 785 nm continuous wave excitation laser. Intensities were normalized to the PL intensity of the 950 nm QW calibration sample.

The gain membrane structure shown in Figure 2 was epitaxially grown with a molecular beam epitaxy VG V80 machine. The growth methods required to grow the semiconductor alloys used in this structure are quite well-known, but the fine-tuning of emission wavelength for a broadband laser differs slightly from what is typical in the growth process of a conventional semiconductor laser. We chose a calibration method in which we first grew a PL sample for both of the QW types, which contained only a single QW group surrounded by barriers. We then fine-tuned the material compositions of each of the QW types separately based on those results and then combined them in the final structure. Figure 3 shows the final structure’s PL curve compared to the PL curves of both of the PL calibration samples. The final structure’s peak PL emission wavelengths hit their targets, as based on previous results reported in this wavelength range,^11^ the laser performance maximum could be expected to red-shift 15 to 25 nm during operation. Since only one QW group was grown into the calibration samples and the final structure consists of a total of 11 QW groups, the much higher PL signal intensity of the final device seen in Figure 3 was anticipated. We may thus conclude from these results that the final structure’s PL signal is as expected based on the PL curves measured from the two QW calibration samples.

From the QW calibration samples’ PL measurements shown in Figure 3, we may also notice that the 950 nm QWs emit roughly only half as strong PL signal compared to the 1010 nm QWs. This difference is due to the reduced quantum confinement in the 950 nm QWs compared to the 1010 nm QWs, as mentioned in the previous chapter. However, since the relative difference in the peak PL intensities between the two wavelength peaks in the final device is only ∼25% and the signal from the shorter wavelength QWs is actually slightly better, we may conclude that the difference in quantum confinement has been well accounted for with the QW amount distribution. Finally, if we compare our PL measurement results shown in Figure 3 to the PL results shown by Leinonen et al.,^11^ we can also assume that the EBLs are indeed preventing the longer wavelength QWs from attracting most of the charge carriers, as we are receiving a proper PL signal from the shorter wavelength QWs as well.

## Laser Characterization

The characterization setup and measurements are described in detail in the following chapter. All results presented were received by measuring at the same heat sink temperature (20 °C). For all measurements, the laser cavity configuration and the laser cavity mirrors remained unchanged.

### Experimentation Setup

**Figure 4:**
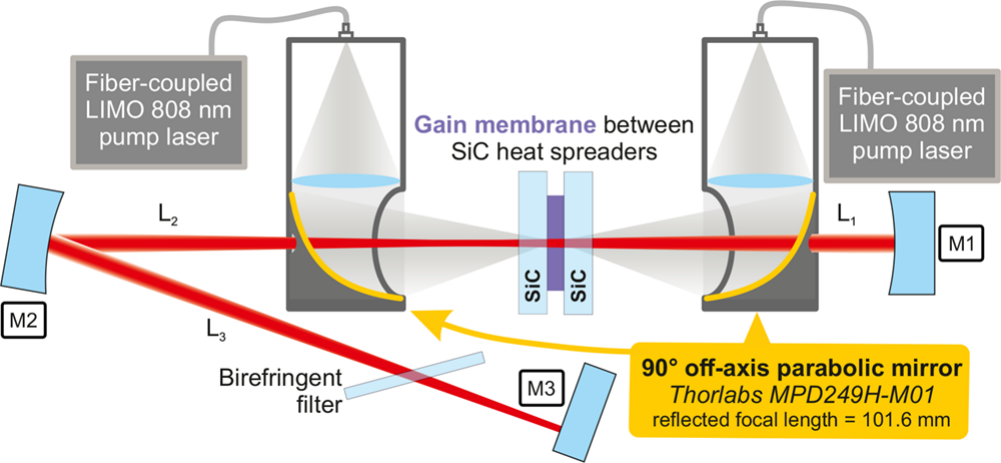
Experimental laser setup utilizing a V-cavity with the broadband gain structure located between mirrors M1 and M2. To focus the pump beams onto the laser-active membrane, parabolic 90° off-axis mirrors featuring a protected gold coating were employed, ensuring a nearly circular pump spot on the gain membrane.

The characterization setup is shown in Figure 4 as a schematic illustration. The characterization cavity was V-shaped, consisting of an outcoupling mirror M3 (plane, reflectivity *R*_M3_ = (99.5 ± 0.3)%) and two mirrors M1 and M2, which both had a radius of curvature of *r*_M1,M2_ = 300 mm and a high reflectivity of *R*_M1,M2_ > 99.9%. The distances *L*_1_ and *L*_2_ between the gain membrane sandwich and the corresponding mirrors M1 and M2 were adjusted to *L*_1_ = 291 mm and *L*_2_ = 293 mm, respectively. The position of mirror M3 was adjusted to create a half opening angle of approximately 5.5° between *L*_2_ and *L*_3_ and a distance of *L*_3_ = 291 mm between mirrors M2 and M3. The laser’s wavelength adjustments were done by using a birefringent filter with a 1 mm thickness. It was positioned under Brewster’s angle within the *L*_3_ resonator arm and oriented to favor parallel polarization. The free spectral range of the birefringent filter was calculated to be 111.2 nm at a wavelength of 988 nm. By applying the transfer matrix method for a Gaussian TEM_00_ beam, the cavity mode diameter on the gain membrane was determined to be approximately 250 μm. As a pump source, a pair of nearly identical (just one manufacturing number between them) LIMO diode lasers operating at 808 nm were employed. Two identical multimode fibers (MHP200L02 from THORLABS), featuring a numerical aperture of 0.22 and a 200 μm core diameter, were used for coupling the pump light. Antireflection coated plano-convex lenses with a focal length of *f* = 100 mm were used to collimate the fiber output. Then, the pump beams were focused on an approximately 330 μm diameter pump spot by 90° off-axis mirrors (shown in Figure 4). The reflectivity of these parabolic mirrors was *R*_808nm_ > 96% due to a protected gold coating. Their reflected focal length was 101.6 mm. Hence, the ratio of the cavity mode to the pump spot diameter stood at approximately 0.76. This value falls within the optimal range of 0.65 to 0.82 as identified by Laurain et al.^24^ through simulations. The parabolic mirrors utilized had a diameter of 50.8 mm alongside a 3 mm diameter hole positioned in its center to avoid intersecting the intracavity laser mode. The collimated pump beam nearly filled the entirety of this mirror’s surface area. Investigations into the losses induced by this 3 mm hole in the pump beam revealed them to be below 1%, rendering them negligible. However, *P*_pump_, the incident pump power, was determined by measuring the reflected power from the parabolic off-axis mirror. Therefore, losses due to fiber coupling, the centered 3 mm through-hole and all reflections, were considered. With the pump laser’s angle of incidence ranging 0° < α_pump_ < 15°, this pumping approach facilitates a close to circular pump spot, characterized at the focus by a sagittal to tangential diameter ratio of *D*_p,sag_/*D*_p,tan_ > 0.96. The gain element of the MECSEL, composed of a gain membrane sandwiched between transparent SiC heat spreaders, was situated in-between two glycol/water-cooled copper heat sinks (not depicted in Figure 4). These heat sinks featured an aperture with a diameter of 1.5 mm and an aperture’s opening angle of 60° to accommodate the incident pump laser adequately (more details can be found elsewhere^25^).

**Figure 5:**
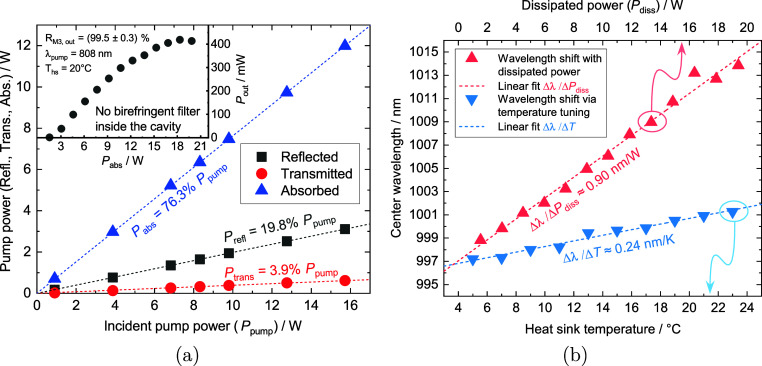
(a) Distribution of incident pump power *P*_pump_ to reflected, transmitted, and absorbed power. (inset) Power transfer measurement under the DSP condition to determine the spectral shift Δλ per absorbed pump power *P*_abs_. (b) Spectral shift, which varies with power, depicted here was obtained during the power transfer measurement illustrated in the inset of Figure 5a.

The thermal resistance *R*_th_ represents one of the most important parameters of MECSELs, as it allows for the comparison and classification of results. In order to calculate *R*_th_, one needs to determine the reflected pump power *P*_refl_, transmitted pump power *P*_trans_, and absorbed pump power *P*_abs_. A power transfer measurement has to be performed to receive the wavelength shift Δλ/Δ*P*_abs_ and the corresponding output power *P*_out_. Also, a temperature tuning measurement is essential to determine the thermal shift, Δλ/Δ*T*_hs_. First, *P*_refl_ was calculated. For 808 nm, the refractive index of SiC is *n*_SiC_ = 2.60.^26^ For angles of incidence between 0 and 15°, the reflectivity of the SiC-air interface changes only in the range of 10^–5;^ therefore, a constant reflection of 19.8% can be assumed for unpolarized light. Second, the linear fit to the transmitted power values revealed a transmission of *P*_trans_ = 3.9%. Then, *P*_abs_ could be calculated: *P*_abs_ = *P*_pump_ – *P*_refl_ – *P*_trans_ = 76.3%. In Figure 5a, it is plotted how the fractions of *P*_pump_ in percent were distributed when interacting with the gain sandwich. The wavelength shift per dissipated power Δλ/*P*_diss_ = (0.90 ± 0.03) nm/W was extracted from the spectra, which were recorded while the power transfer behavior was measured (shown in the inset of Figure 5a). Δλ/*P*_diss_ is plotted over dissipated power *P*_diss_ in Figure 5b. *P*_diss_ was calculated as follows: *P*_diss_ = *P*_abs_ – *P*_out_, where *P*_out_ is the output power measured. Considering the relatively high outcoupler reflectivity, *R*_M3,out_ = (99.5 ± 0.3)%, high-power laser emission was not anticipated. However, we observed the output power *P*_out_ increasing linearly (plotted in the inset of Figure 5a), commencing at the threshold power of *P*_th_ = 1.63 W. A slight deviation from this linear behavior is noticeable at approximately 11 W of absorbed pump power *P*_abs_. This deviation may arise from a polarization shift, given the absence of a polarization-maintaining component such as a birefringent filter.

Apart from the slope efficiency change, no deviation or change from the usual linear trend in the spectral shift (refer to Figure 5b) is apparent. The experimental setup for this measurement was the same as that depicted in Figure 4. No optical intracavity elements, such as a birefringent filter, were employed in this power measurement—the laser was operated freely. *P*_pump_ was symmetrically increased in the double-side configuration. Lastly, the thermal dependence of the laser emission, expressed as Δλ/Δ*T*_hs_, was assessed under a constant incident pump power of *P*_pump_ = 6 W (3 W per side), while the heat sink temperature *T*_hs_ was gradually adjusted from 5 to 23 °C by altering the glycol/water coolant temperature. The temperature variance between the heat sink and the chiller was found to reside within a tolerance of 0.1 °C, rendering it negligible. The thermal wavelength shift, represented as Δλ/Δ*T*_hs_ = (0.24 ± 0.01) nm/K, was determined through linear regression applied to the data plotted in Figure 5b. This enabled the determination of the MECSEL gain element’s thermal resistance *R*_th_, incorporating error propagation as per Heinen et al.^27^ This was achieved by dividing the spectral shift per *P*_diss_ by the spectral shift per change in *T*_hs_, yielding *R*_th_ = (3.75 ± 0.28) K/W. Taking into account the utilization of SiC heat spreaders (approximately 350 μm in thickness each), this outcome aligns with the anticipated range as per Phung et al.^23^ and mirrors a prior finding of our own earlier work.^28^

### Broadband Wavelength Tuning

**Figure 6:**
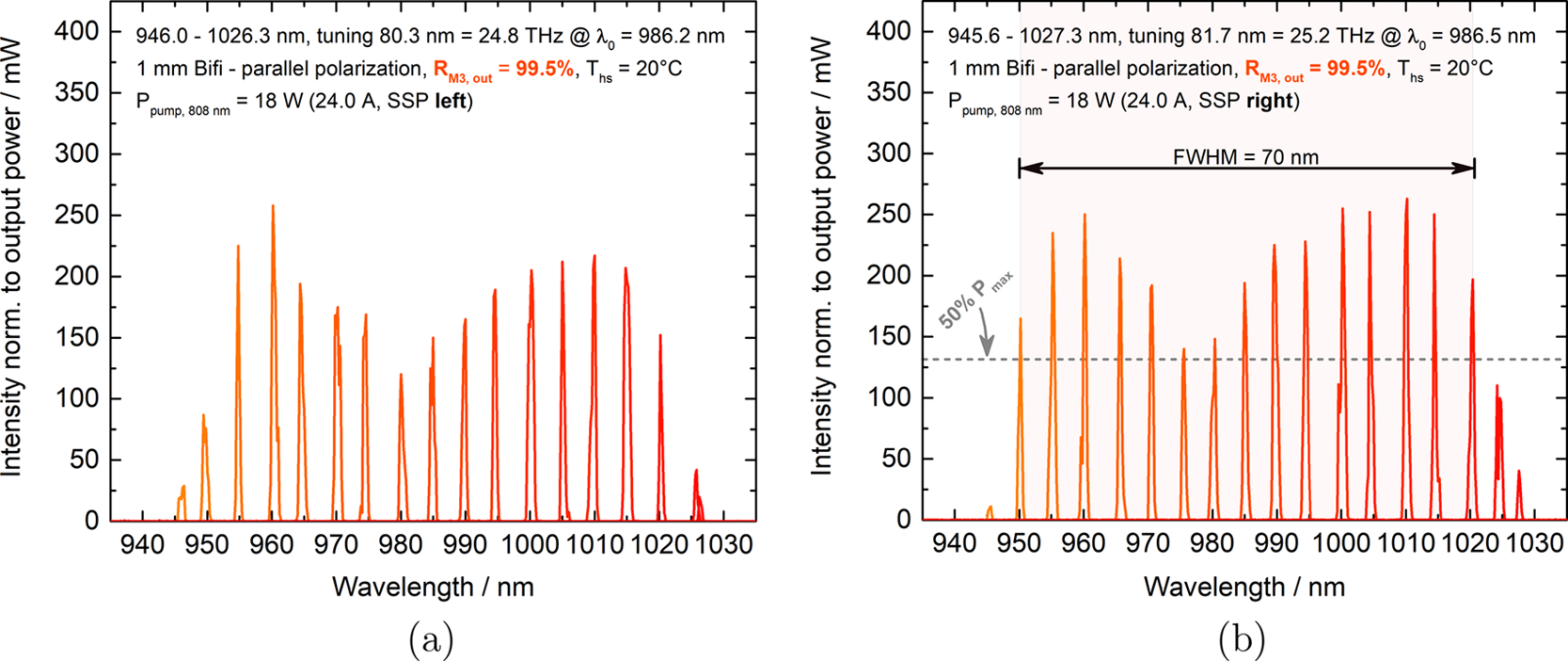
Two plots of an exemplary set of spectra (orange → red curves) depicting wavelength tuning are presented here. The spectral intensities were normalized to the measured output power and plotted over wavelength correspondingly. The 18 W of pump power were irradiated (a) from the left and (b) from the right side only.

For tuning measurements, an incident pump power of *P*_pump_ = 18.0 W was chosen, because our preliminary tests suggested that this pump power would result in the largest tuning range when using the SSP regime. Any higher pump powers led to thermal rollover under the SSP condition, which, as a result, led to a reduced tuning width and overall inferior output power. The outer heat spreader facets of the gain element were antireflection coated for the wavelengths of the laser as well as the pump to reduce cavity losses. The spectra were recorded with an ANDO AQ-6315A optical spectrum analyzer, which had a resolution limit of 0.05 nm. Figure 6a,b shows the results of the SSP tuning measurements. As described in the section “Design and Structure Simulation”, we can see a slightly better performance in both output power and tuning range with the right side SSP situation (Figure 6b). This is due to a favorable distribution of barrier widths. Similar kinds of structures with comparable results have been shown for example by Baumgärtner et al.^29^ and Phung et al.^30^ Additionally, when pumping from the right, the output power did not drop below 50% of *P*_max_ between the performance peaks of the two different kinds of QWs and a FWHM of 70 nm was reached while maintaining an output power of >125 mW. For the right side pumping in Figure 6b, the output power near the longer wavelength range around 1005 nm is similar to the shorter wavelength around 960 nm. For the left side pumping (Figure 6a), there is a clear difference, as the output power near the longer wavelength range is about 25% inferior to the shorter wavelength range. This finding clearly indicates the significance of carefully taking into account the absorption behavior of the pump light inside the structure when determining the distributions of barrier and QW widths in future gain structure designs.

**Figure 7:**
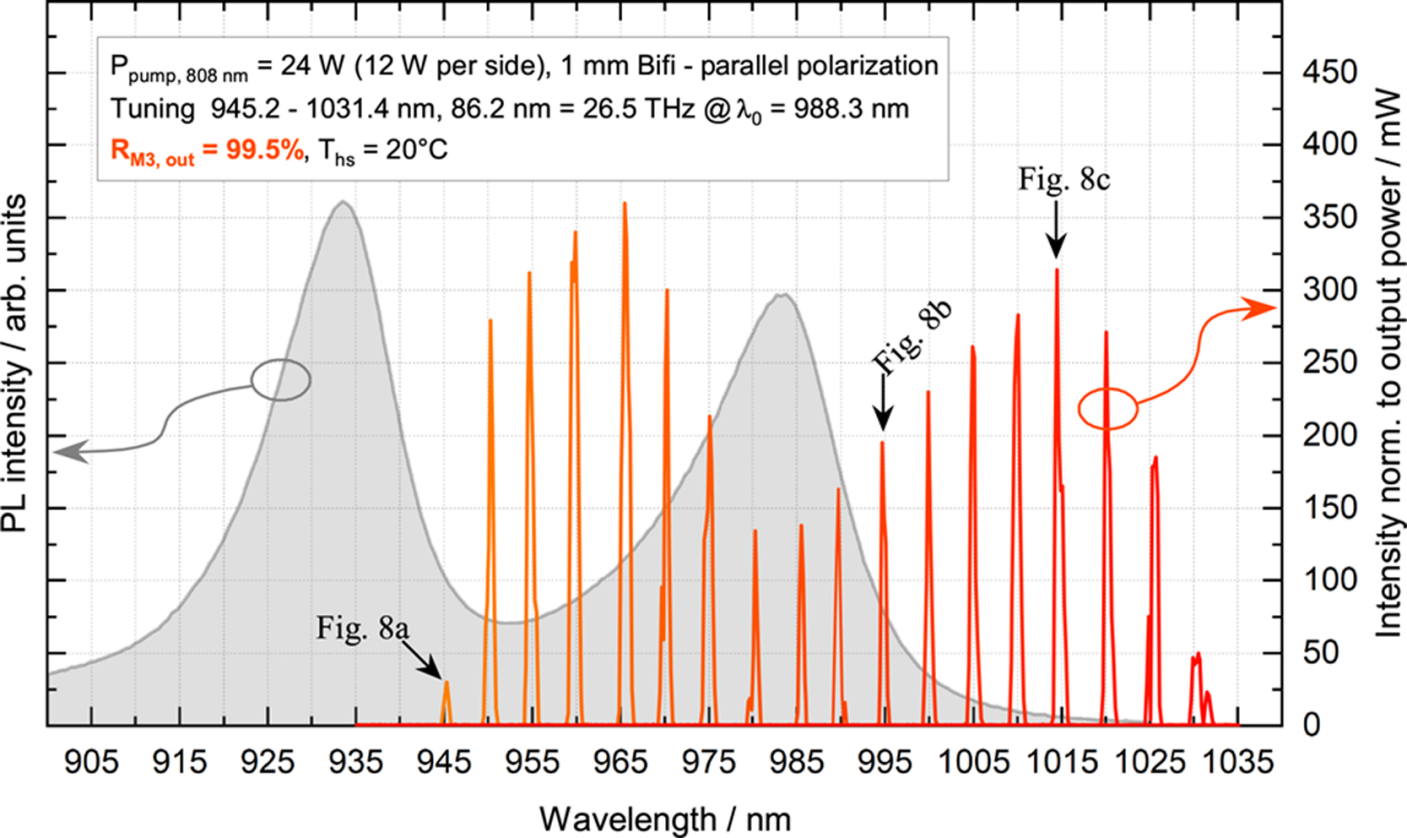
Exemplary spectra (orange → red curves) depicting the wavelength tuning under symmetric DSP conditions are shown in this plot. The spectral intensities were normalized based on their corresponding measured output powers, and the results were plotted against wavelength. The PL (gray curve with filled area underneath) of the corresponding broadband gain structure (see Figure 3) plotted for comparison with the MECSEL’s tuning characteristic.

In order to obtain a maximum full tuning width, the total incident pump power *P*_pump_ was increased to 24 W and divided equally to 12 W per side when applying the DSP regime. Beforehand experiments (see Figure 5a) revealed that the best output power performance and the largest tuning range under DSP conditions will be reached with this pump power configuration. As seen in Figure 7, a tuning range of 86.2 nm or 26.5 THz was achieved, which is wider than with the SSP measurements, where a tuning range of ∼80 nm was reached. However, since the peak output powers received at the 965 and 1015 nm rose significantly when applying DSP, the FWHM tuning range is no longer as wide, as the output power in the middle of the spectrum is just barely under half of the peak power. If, however, we look at the tuning range at which 125 mW of output power (which was roughly the FWHM output power level in the SSP case) is maintained, we have an outstanding tuning range of 80 nm or 25 THz reaching from 947 to 1027 nm.

To highlight the connection, the PL spectrum of the broadband gain structure (already shown in Figure 3) was plotted with laser tuning in Figure 7. It is particularly notable how closely the tuning behavior of the MECSEL conforms to the PL characteristic when comparing relative intensities, and how clearly the spectra show the spectral red shift and the typical inverted parabolic shape of the gain of the corresponding QWs.

### Beam Profiles

**Figure 8:**
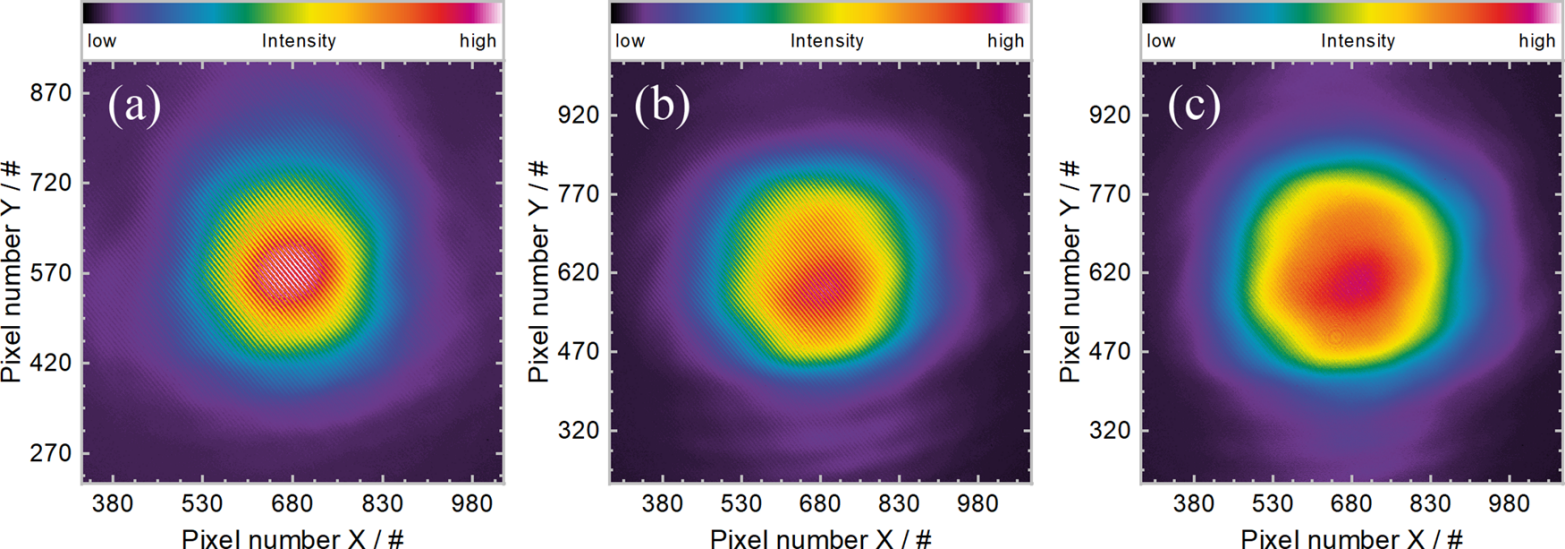
Beam profiles taken during the tuning measurement are plotted in Figure 7. Three examples have been chosen at (a) low (∼945 nm), (b) medium (∼995 nm), and (c) high (∼1015 nm) output power.

It is a built-in feature of MECSELs to possess the same excellent beam quality properties (*M*^2^ < 1.1 and a TEM_00_ Gaussian transverse mode profile^10,28^) as VECSELs.^31,32^ This is enabled by satisfying the geometrical condition of having the squared gain region thickness *L*^2^ much smaller than the laser mode area *s* (*L*^2^ ≪ *s*) inside the laser cavity.^33^ In this work, the relation is 1:12,272 with *L*^2^ = 4 μm^2^ and *s* = 49,087 μm^2^. The external mirrors provide full mode control as the gain region’s impact on beam distortion is present,^28^ but minimal compared to classical solid-state lasers. Therefore, the behavior of the transverse mode shape during the tuning measurements is an important characteristic to prove undisturbed operation of the laser. Beam profiles were recorded every second that a spectrum was taken. In Figure 8, three exemplary beam profiles for low, medium, and high output power were plotted. In the plot of Figure 7, it is indicated to which spectrum the plotted beam profiles of Figure 8 correspond. Small deformations of the beam profile can be seen in Figure 8b,c, but the fundamental Gaussian TEM_00_ character remains intact. During beam profile recording and taking spectra with the optical spectrum analyzer, the laser cavity was not realigned and all parameters were kept constant, solely the birefringent filter was rotated. This shows that the excellent beam properties typical to MECSELs were maintained with the novel multitype QW design.

## Conclusions

**Figure 9:**
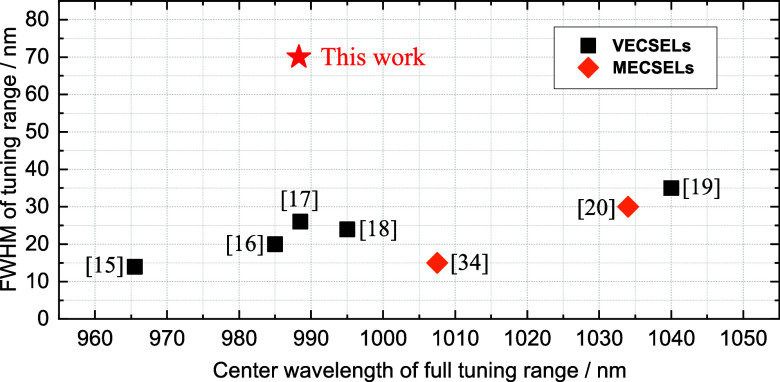
Overview of the FWHM of tuning ranges of a selection of VECSELs (black squares) and MECSELs (full orange rhombuses) with reportedly wide full tuning ranges around 1000 nm emission wavelength plotted over their central emission wavelength of their FWHM tuning ranges. Both QW- and QD-based laser active structures are included.

The broadest tuning range of a semiconductor laser with vertical emission around 1 μm, namely, 26.5 THz (86.2 nm), was shown. Of greater importance is the FWHM tuning of 70 nm (with at least 125 mW of output power), which is effectively double that of previously reported tuning results in the 1 μm wavelength range. This wavelength range was chosen because there are several notable benchmarks for a wide tuning range and because the material system used is very well developed and therefore does not introduce any additional variables. Figure 9 summarizes the development of tuning bandwidths in the 1 μm wavelength range by showing the advances made with VECSELs and MECSELs and how the presented work compares to them. The comparison is made here by taking into account the FWHM of the emission spectrum, as this is ultimately decisive if we want to have a constant power over the tuning range.

It is worth noting that the VECSELs seen in Figure 9 were explicitly designed for wide tuning, while the MECSELs were not, also due to their relative novelty in the field (reference Mirkhanov et al.^34^ was added for comparison), which already shows the potential and inherent advantage when it comes to tuning range, as demonstrated with Figure 1. We can further see from Figure 9 that when it comes to FWHM of the tuning range, our design utilizing multiple types of QWs is a highly effective strategy that does not rely on complicated processing, coatings, or pumping setups but just optimized epitaxial design of the gain structure. The results demonstrate the remarkable potential of MECSELs employing many types of QWs to greatly expand the tuning range of vertically emitting semiconductor lasers, while maintaining excellent beam quality (*M*^2^ < 1.1) and high power (>125 mW) throughout the whole tuning range. In our design process, four relevant strategy points were recognized: (1) separation of different types of QW areas inside the active region from each other with EBLs to maintain sufficient carrier concentration in all QWs, (2) ensuring maximum overlap between the standing wave of the optical field and the QWs on their designed emission wavelengths, while minimizing the overlap with other QW types, if they are capable of absorbing the photons emitted, (3) choosing correct QW materials and particularly the nominal emission wavelength between them, and (4) designing the structure as a whole so that each of the QW types are pumped equally.

This paper has only scratched the surface and shown the applicability when it comes to the potential of multitype QW MECSELs. The advances that can be made from this point onward are 2-fold. First of all, more QW types could be utilized either to increase the emission power in the middle of the tuning range if more power throughout the tuning range is needed or to widen the tuning range by choosing three or more QW types accordingly. Secondly, and more importantly, the total thickness of the MECSEL could be at least doubled to truly take advantage of the MECSELs’ double-side pumping capabilities, when we keep in mind that the structure shown in this paper could still be properly pumped from one side only. The doubling of the total thickness allows for many more QW groups to be utilized, which in turn can then be turned into either higher emission power or wider tuning range. In the DSP regime, two pump lasers operating at different wavelengths could also be used to cover a wider pumping wavelength range and avoid absorption in unwanted regions of the active area. Finally, stacking of more than one MECSEL with additional heat spreaders in-between instead of complete monolithic structures is a great option for future widely tunable multitype QW MECSELs.

## Notes

A preprint version of this work was published in arXiv.^35^
